# Supplementary material for: Nonsense mutations can increase mRNA levels
Source: Biol Open. 2026 Apr 27;15(4):bio062444. doi: 10.1242/bio.062444 (PMC13225217; doi:10.1242/bio.062444)
Supplement: Supplementary information [file biolopen-15-062444-s1.pdf]

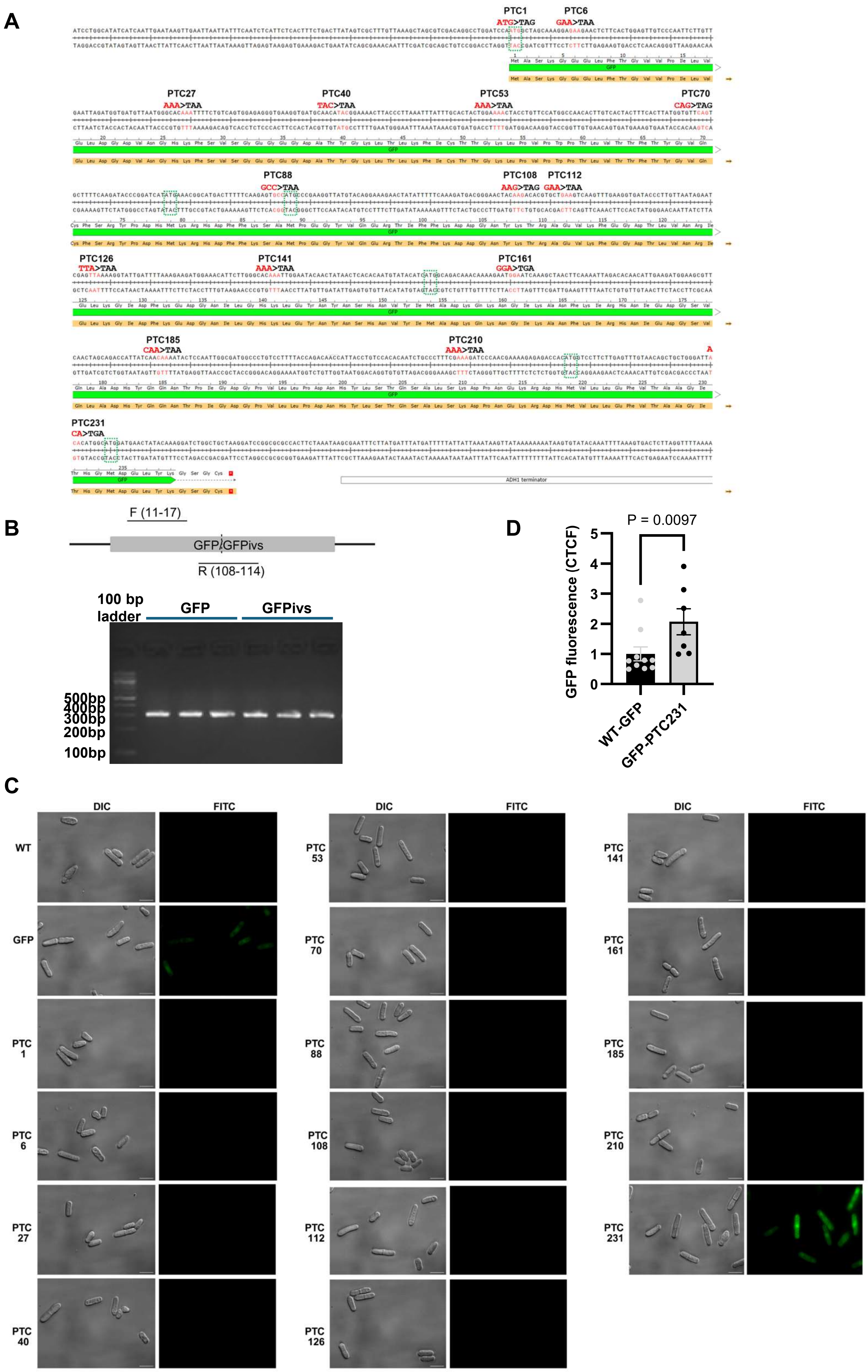

Biology Open • Supplementary information

Fig. S1. Characterisation of reporters. A) GFP coding sequence. Nonsense codon positions are indicated in red. The codons in each case were changed to a PTC using site-directed mutagenesis. The original codons and their corresponding PTC substitution are shown. The sequences highlighted in the green-dotted boxes show the ATG codons in GFP. The image was generated in SnapGene Viewer. **B)** Validation of the NMD qRT-PCR primers. PCR was performed under saturating conditions (40 cycles) to increase the chance of detecting any pre-mRNA or unspliced transcripts using three independent WT-GFP and WT-GFPivs cDNAs, and to verify that the qRT-PCR primers (GFP-qPCR-F and GFP-qPCR-R, sequences in Supplementary Table 2) were specific for the spliced transcript (in this case, GFP). Both primer pairs work with GFP and GFPivs, since during splicing the intron is removed, producing only spliced GFP with a cDNA similar to that of WT-GFP. The approximate positions of the qPCR primers in GFP are shown. The dotted line in the schematic above the gel signifies the splice site. A 1% agarose gel was used. **C)** Representative DIC and fluorescence (FITC) images of the WT, GFP, and GFP-PTC-containing strains. Scale bars = 10μm. **D)** Image quantification of the GFP and GFP-PTC231 cells from C. 10 WT-GFP and 7 GFP-PTC231 independent cultures were imaged, and the corresponding images were quantified using ImageJ to compare GFP fluorescence. The mean fluorescence values are shown. Error bars represent the standard error of the mean across replicates. In each image, four individual cells were quantified, totalling 40 cells for WT-GFP and 28 for GFP-PTC231. Mann-Whitney rank sum test was used to test for statistical significance.

Biology Open • Supplementary information

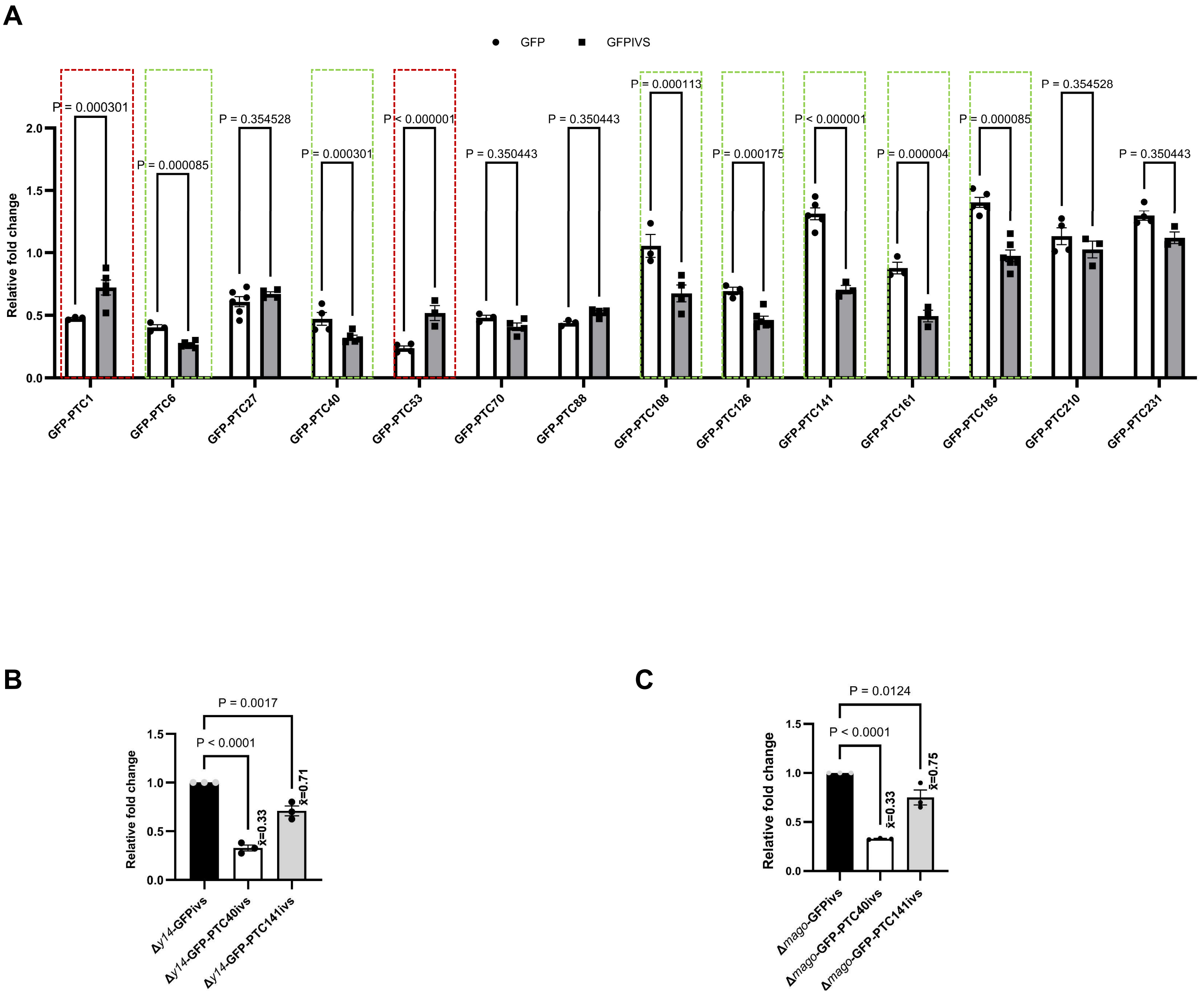

Biology Open • Supplementary information

Fig. S2. Splicing-dependent NMD does not require *Y14* and *MAGO*. A) Pairwise comparison of GFP and GFPivs transcript levels. Error bars represent the standard error of the mean across replicates (n≥3). The pairwise comparisons highlighted in red represent instances where a significant increase in mRNA levels was observed in the spliced constructs compared to the intronless constructs. The comparisons highlighted in green represent instances where splicing enhances NMD. The unhighlighted comparisons indicate cases where no significant differences were observed. A t-test was used to test for statistical significance. **B)** qRT-PCR comparing transcript levels of *Δy14*-GFPivs, *Δy14*-GFP-PTC40ivs, and *Δy14*-GFP-PTC141ivs. Transcript levels were normalised to *Δy14*-GFPivs, with *rpl32* serving as an internal control. Error bars represent the standard error of the mean values between replicate experiments (n=3). A one-way ANOVA with Dunnett's multiple comparisons was used to test for statistical significance (**$\bar{x}$=** mean fold change value)**. C)** qRT-PCR comparing transcript levels of *Δmago*-GFPivs, *Δmago*-GFP-PTC40ivs, and *Δmago*-GFP-PTC141ivs. Transcript levels were normalised to *Δmago*-GFPivs, with *rpl32* serving as an internal control. Error bars represent the standard error of the mean values between replicate experiments (n=3). A one-way ANOVA with Dunnett's multiple comparisons was used to test for statistical significance (**$\bar{x}$=** mean fold change value).

Biology Open • Supplementary information

Table S1. Yeast strains used in this study

Strain	Genotype	Source
WT-pDUAL-GFP	h+ *leu1*-32 *ura4*DS/E pDUAL-GFP	Wen and Brogna, 2010
WT-pDUAL-GFP PTC1	h+ *leu1*-32 *ura4*DS/E pDUAL-GFP-PTC1	This study
WT-pDUAL-GFP-PTC6	h+ *leu1*-32 *ura4*DS/E pDUAL-GFP-PTC6	Wen and Brogna, 2010
WT-pDUAL-GFP-PTC27	h+ *leu1*-32 *ura4*DS/E pDUAL-GFP-PTC27	Wen and Brogna, 2010
WT-pDUAL-GFP-PTC40	h+ *leu1*-32 *ura4*DS/E pDUAL-GFP-PTC40	This study
WT-pDUAL-GFP-PTC53	h+ *leu1*-32 *ura4*DS/E pDUAL-GFP-PTC53	This study
WT-pDUAL-GFP-PTC70	h+ *leu1*-32 *ura4*DS/E pDUAL-GFP-PTC70	This study
WT-pDUAL-GFP-PTC108	h+ *leu1*-32 *ura4*DS/E pDUAL-GFP-PTC108	This study
WT-pDUAL-GFP-PTC112	h+ *leu1*-32 *ura4*DS/E pDUAL-GFP-PTC112	This study
WT-pDUAL GFP-PTC126	h+ *leu1*-32 *ura4*DS/E pDUAL-GFP-PTC126	This study
WT-pDUAL-GFP-PTC141	h+ *leu1*-32 *ura4*DS/E pDUAL-GFP-PTC141	This study
WT-pDUAL-GFP-PTC161	h+ *leu1*-32 *ura4*DS/E pDUAL-GFP-PTC161	This study
WT-pDUAL-GFP-PTC185	h+ *leu1*-32 *ura4*DS/E pDUAL-GFP-PTC185	This study
WT-pDUAL-GFP-PTC210	h+ *leu1*-32 *ura4*DS/E pDUAL-GFP-PTC210	This study
WT-pDUAL-GFP-PTC231	h+ *leu1*-32 *ura4*DS/E pDUAL-GFP-PTC231	This study
WT-pDUAL-GFPivs	h+ *leu1*-32 *ura4*DS/E pDUAL-GFPivs	This study
WT-pDUAL-GFP-PTC1ivs	h+ *leu1*-32 *ura4*DS/E pDUAL-GFP-PTC1ivs	This study
WT-pDUAL-GFP-PTC6ivs	h+ *leu1*-32 *ura4*DS/E pDUAL-GFP-PTC6ivs	This study
WT-pDUAL-GFP-PTC27ivs	h+ *leu1*-32 *ura4*DS/E pDUAL-GFP-PTC27ivs	This study
WT-pDUAL-GFP-PTC40ivs	h+ *leu1*-32 *ura4*DS/E pDUAL-GFP-PTC40ivs	This study
WT-pDUAL-GFP-PTC53ivs	h+ *leu1*-32 *ura4*DS/E pDUAL-GFP-PTC53ivs	This study
WT-pDUAL-GFP-PTC70ivs	h+ *leu1*-32 *ura4*DS/E pDUAL-GFP-PTC70ivs	This study
WT-pDUAL-GFP-PTC88ivs	h+ *leu1*-32 *ura4*DS/E pDUAL-GFP-PTC88ivs	This study
WT-pDUAL-GFP-PTC108ivs	h+ *leu1*-32 *ura4*DS/E pDUAL-GFP-PTC108ivs	This study
WT-pDUAL-GFP-PTC126ivs	h+ *leu1*-32 *ura4*DS/E pDUAL-GFP-PTC126ivs	This study
WT-pDUAL-GFP-PTC141ivs	h+ *leu1*-32 *ura4*DS/E pDUAL-GFP-PTC141ivs	This study

Table S1 continued: Yeast strains used in this study

Strain	Genotype	Source
WT-pDUAL-GFP-PTC161ivs	h+ *leu1*-32 *ura4*DS/E pDUAL-GFP-PTC161ivs	This study
WT-pDUAL-GFP-PTC185ivs	h+ *leu1*-32 *ura4*DS/E pDUAL-GFP-PTC185ivs	This study
WT-pDUAL-GFP -PTC210ivs	h+ *leu1*-32 *ura4*DS/E pDUAL-GFP-PTC210ivs	This study
WT-pDUAL-GFP-PTC231ivs	h+ *leu1*-32 *ura4*DS/E pDUAL-GFP-PTC231ivs	This study
Δ*upf1*-pDUAL-GFP	h+ *ade6*-210 *arg3*D *his3*D *leu1*-32 *ura4*DS/E *upf1*:kanMX6 pDUAL-GFP	This study
Δ*upf1*-pDUAL-GFP-PTC6	h+ *ade6*-210 *arg3*D *his3*D *leu1*-32 *ura4*DS/E *upf1*:kanMX6 pDUAL-GFP-PTC6	This study
Δ*upf1*-pDUAL-GFP-PTC141	h+ *ade6*-210 *arg3*D *his3*D *leu1*-32 *ura4*DS/E *upf1*:kanMX6 pDUAL-GFP-PTC141	This study
Δ*upf1*-pDUAL-GFP-PTC40ivs	h+ *ade6*-210 *arg3*D *his3*D *leu1*-32 *ura4*DS/E *upf1*:kanMX6 pDUAL-GFP-PTC40ivs	This study
Δ*upf1*-pDUAL-GFP-PTC141ivs	h+ *ade6*-210 *arg3*D *his3*D *leu1*-32 *ura4*DS/E *upf1*:kanMX6 pDUAL-GFP-PTC141ivs	This study
Δ*mago*-pDUAL-GFPivs	h- *leu1*-32 *ura*D4-D18, *mago*del:clonatR pDUAL-GFPivs	This study
Δ*mago*-pDUAL-GFP-PTC40ivs	h- *leu1*-32 *ura*D4-D18, *mago*del:clonatR pDUAL-GFP-PTC40ivs	This study
Δ*mago*-pDUAL-GFP-PTC141ivs	h- *leu1*-32 *ura*D4-D18, *mago*del:clonatR pDUAL-GFP-PTC141ivs	This study
Δ*y14*-pDUAL-GFPivs	h- *leu1*-32 *ura*D4-D18, *y14*del:clonatR pDUAL-GFPivs	This study
Δ*y14*-pDUAL-GFP-PTC40ivs	h- *leu1*-32 *ura*D4-D18, *y14*del:clonatR pDUAL-GFP-PTC40ivs	This study
Δ*y14*-pDUAL-GFP-PTC141ivs	h- *leu1*-32 *ura*D4-D18, *y14*del:clonatR pDUAL-GFP-PTC141ivs	This study
Flag-Rpb3-WT-pDUAL-GFP	h- *leu1*-flag-rpb3 *ade*6-M216 *ura* D18 pDUAL-GFP	This study
Flag-Rpb3-WT-pDUAL-GFPivs	h- *leu1*-flag-rpb3 *ade*6-M216 *ura* D18 pDUAL-GFPivs	This study

Biology Open • Supplementary information

Table S2. Primers used in this study

Primer name	Description	Sequence
Ubc4_F	*Ubc4* cloning	CAAAGATGACGGGAACTACAAGACACGTAT GTAACTATTTAGTCTTGTGTAGATTATG
Ubc4_R	*Ubc4* cloning	GTATCACCTTCAAACTTGACTTCAGCACCTA CGAATATGCTGTTAGTTATC
GFP-qPCR-F	qPCR	GAGTTGTCCCAATTCTTGTT
GFP-qPCR-R	qPCR	CTTGACTTCAGCACGTGTCTT
PTC1 F	PTC1 mutagenesis	GGATCCATAGGCTAGCAAAGGAGAAGAACT CTTCACTGGAGTTGTCCCAATTCTTG
PTC1 R	PTC1 mutagenesis	GCTAGCCTATGGATCCAGGCCTGTCGACG CTAGCTTTAACAAAGCGACTATAAGTC
PTC40_F	PTC40 mutagenesis	GATGCAACATAAGGAAAACTTACCCTTAAAT TTATTTGCACTACTGGAAAACTACCTG
PTC40_R	PTC40 mutagenesis	GTTTTCCTTATGTTGCATCACCTTCACCCTC TCCACTGACAGAAAATTTG
PTC53_F	PTC53 mutagenesis	CTACTGGATAACTACCTGTTCCATGGCCAA CACTTGTCACTACTTTCAC
PTC53_R	PTC53 mutagenesis	CAGGTAGTTATCCAGTAGTGCAAATAAATTT AAGGGTAAGTTTTCCGTATGTTGCATC
PTC70_F	PTC70 mutagenesis	CTTATGGTGTTTAGTGCTTTTCAAGATACCC GGATCATATGAAACGGCATGAC
PTC70_R	PTC70 mutagenesis	CTTGAAAAGCACTAAACACCATAAGTGAAA GTAGTGACAAGTGTTGGCCATGGAAC
PTC88_F	PTC88 mutagenesis	CAAGAGTTAAATGCCCGAAGGTTATGTACA GGAAAGAACTATATTTTTCAAAGATGAC
PTC88_R	PTC88 mutagenesis	TCGGGCATTTAACTCTTGAAAAAGTCATGC CGTTTCATATGATCCGGGTATC
PTC108_F	PTC108 mutagenesis	GAACTACTAGACACGTGCTGAAGTCAAGTT TGAAGGTGATACCCTTGTTAATAG
PTC108_R	PTC108 mutagenesis	ACGTGTCTAGTAGTTCCCGTCATCTTTGAAA AATATAGTTCTTTCCTGTACATAACCTTC
PTC_112_F	PTC112 mutagenesis	CACGTGCTTAAGTCAAGTTTGAAGGTGATA CCCTTGTTAATAGAATCGAGTTAAAAG
PTC_112_R	PTC112 mutagenesis	CTTGACTTAAGCACGTGTCTTGTAGTTCCC GTCATCTTTGAAAAATATAGTTCTTTCCTG
PTC126_F	PTC126 mutagenesis	GTTAATAGAATCGAGTAAAAAGGTATTGATT TTAAAGAAGATGGAAACATTCTTGGGCAC
PTC126_R	PTC126 mutagenesis	CAATACCTTTTTACTCGATTATATTAACAAG GGTATCACCTTCAAACTTGACTTCAG
PTC_161_F	PTC161 mutagenesis	CAAAAGAATTGAATCAAAGCTAACTTCAAAA TTAGACACAACATTGAAGATGGAAG
PTC_161_R	PTC161 mutagenesis	CTTTGATTCAATTCTTTTGTTTGTCTGCCAT GATGTATACATTGTGTGAGTTATAGTTG
PTC_185_F	PTC185 mutagenesis	CATTATCAATAAAATACTCCAATTGGCGATG GCCCTGTCCTTTTACCAGAC

Biology Open • Supplementary information

Table S2 continued: Primers used in this study

Primer name	Description	Sequence
PTC_185_R	PTC_185 mutagenesis	CAATTGGAGTATTTTATTGATAATGGTCTGC TAGTTGAACGCTTCCATCTTCAATGTTG
PTC_210_F	PTC_210 mutagenesis	CTTTCGTAAGATCCCAACGAAAAGAGAGAC CACATGGTCCTTCTTGAGTTTGTAACAGC
PTC_210_R	PTC_210 mutagenesis	GTTGGGATCTTACGAAAGGGCAGATTGTGT GGACAGGTAATGGTTGTCTGGTAAAAGGAC
PTC_231_F	PTC_231 mutagenesis	TGGGATTTGACATGGCATGGATGAACTATA CAAAGGATCTGGCTG
PTC_231_R	PTC_231 mutagenesis	TGCCATGTCAAATCCCAGCAGCTGTTACAA ACTCAAGAAGGACCATG
Rpl32_F	qPCR	TGGATTGAAGGCTTTCCTAGTC
Rpl32_R	qPCR	CAGAGACGTTACCAGCAATCT
PTC108 qPCR_R	qPCR	CTTGACTTCAGCACGTGTCTA
18S F	qPCR	CAATTGGAGGGCAAGTCTGG
18S R	qPCR	GTCGACCAGGCTCAAAGTTC
Intergenic_F	ChIP qPCR	GAA CGA ACC AGG ACG GCT AT
Intergenic_R	ChIP qPCR	TGC ATG GAG GGT TTC CAC TC
ChIP_GFP_(P1)_F	ChIP qPCR	ACGATTACTGGGGAGAGAAAACA
ChIP_GFP_(P1)_R	ChIP qPCR	GTGCCCATTAACATCACCATCT
ChIP_GFP_(P2)_F	ChIP qPCR	GAGTTGTCCCAATTCTTGTTGA
ChIP_GFP_(P2)_R	ChIP qPCR	AGTGAAAGTAGTGACAAGTGTT
ChIP_GFP_(P3)_F	ChIP qPCR	TGCTTTTCAAGATACCCGGATCA
ChIP_GFP_(P3)_R	ChIP qPCR	GCCCAAGAATGTTTCCATCTTCT
ChIP_GFP_(P4)_F	ChIP qPCR	TGTATACATCATGGCAGACAAA
ChIP_GFP_(P4)_R	ChIP qPCR	GGTCTCTCTTTTCGTTGGGATCT
ChIP_GFP_(P5)_F	ChIP qPCR	TCCCAACGAAAAGAGAGACCA
ChIP_GFP_(P5)_F	ChIP qPCR	AGCGACCTCATACTATACCTGA

Biology Open • Supplementary information
